# Drawings to explore faculties‘ and students‘ perceptions from different generations cohorts about dental education: A pilot study

**DOI:** 10.1038/s41405-022-00109-5

**Published:** 2022-06-15

**Authors:** Renata Grazziotin-Soares, Diego Machado Ardenghi

**Affiliations:** College of Dentistry, University of Saskatchewan, Saskatoon, Canada

**Keywords:** Dental clinical teaching, Continuing professional development in dentistry

## Abstract

**Introduction/Aims:**

We aimed at using drawings as a form of data collection to give voice to older and younger generations in regards to educational practices in undergraduate dentistry.

**Materials and methods:**

First year dental students (younger generations) and faculty members (older generations) produced drawings depicting their perceptions of the current dental education learning environment. Qualitative analysis was conducted independently by two researchers using the drawings to produce codes, categories and themes.

**Results:**

15 drawings were produced: 9/34 (26.4%) made by students and 6/20 (30%) made by faculty members. The generated themes indicated that students and faculties found that dental education is going through a challenging time, because of the restrictions caused by the COVID-19 pandemic; and that they were aware about the evident division between basic/preclinical and applied/clinical courses. Faculties showed hopeful signs that the situation may get better. Students‘ drawings evoked the following topics: digital connectedness, diversity, time goes by, and future aspirations in Dentistry.

**Discussion:**

This study reinforced the validity of visual methods as an approach in research and showed different graphical features (features that might be intentionally or unintentionally represented in the drawings) that gave voice to participants. These voices could have been invisible in more traditional qualitative approaches, such as interviews or questionnaires.

**Conclusions:**

Although the two groups of participants came from different generation cohorts, they had aligned perceptions regarding challenges in dental education, and mentioned the separation between preclinic and clinic. Drawings were unique, innovative, and an interesting tool to express perceptions regarding today’s learning environment. These insights can consequently help educators to personalize teaching approaches to better meet the needs of the students.

## Introduction

The concept of ‘generations’ (also called ‘generations-theory’) consists of a set of beliefs centred around the idea that an individual’s values, motivations, and behaviours can be presumed based upon that individual’s date of birth [1]. Generational changes are grounded in shifts in culture and reflect changes in society [2]. Much of contemporary application of generations-theory is based on ideas created by sociologists who developed a generational schema based upon the US history [3, 4]. Their framework on social generations label different cohorts of people, sometimes using stereotypes [5]. Then, the term ‘generation’ may be explained as ‘all the people who were born and live at the same time’, in an average period of 20 to 30 years. These people, collectively, should experience the same significant events within a given period [5, 6].

Apart from the US and Western world history, in other areas, generations of people also receive specific denominations that are framed by their social historical reference points, culturally determined experiences, and individual interpretations [7]. In South Africa, for example, people born after the 1994 general election, the first election after apartheid ended, are often referred as the “Born-free generation” [8] and, in Armenia, people born after the country’s independence, in 1991, are known as the “Independence generation” [9]. As the most researched concept on generations is linked by specific historical and cultural circumstances of US history (such as the ‘Baby Boomers’, who are the people born following World War II or people who may have fought in the Vietnam War), this current paper is using the ‘Western world/US’ definition for generations cohorts (see definitions and characteristics of different cohorts in Table 1).

**Table 1:** Timeline showing different cohorts of generations that participated in this study.

		Who are they?	Characteristics
**Students**	**Generation Z** (1997–2012)	Currently, (at the time of this paper being written) these cohort includes people 9 to ~24 years of age. So, essentially many are in schooling of some sort (elementary, middle school, high school, college/university, or recent college/university graduate).	Technology is part of their life Highly connected Multi-tasking Attentive to inclusion and equity Reduced attention spans Fast decision-making
	**Generation Y, Millennials** (Born on 1981 to 1996 or 2000s)	They grew up around the turn of the 3rd millennium. This cohort surpassed the Baby Boomers in number (in the United States). They are the most formally educated generation.	Digital natives – communicate through technology Confident Team oriented Motived by self-interest Expect immediate feedback
**Faculty members**	**Generation X** (1965–1980)	They are in their middle, high earning years of their careers. This generation is also called ‘Baby bust” because of a drop in birth rates following the previous baby boom period.	Value the balance work & leisure Problem-solvers Independent and self-reliant Competent with technology Comfortable with authority
	**Baby Boomers** (Born between 1946 and 1964)	People born after the World Wars ended. This is a relatively large demographic cohort because of the increased birth rates (“baby boom”). They (also) may have fought in the Vietnam War or participated in the counterculture of the 1960s	Read printed books and use handouts Judgmental Networking, interactives Like ‘easy-to-scan’ information (material traditionally organized)

The table concepts based on the ‘Western world/US generations’ [Strauss–Howe generational theory [3, 46]*, (Statistics Canada [https://www12.statcan.gc.ca/census-recensement/2011/as-sa/98–311-x/98–311-x2011003_2-eng.cfm]), and some characteristics/styles of each generation-learner [21, 45]*.

*Specific initial and end dates are variable depending on the source.

In higher education health science fields, generations-theory provides a logical reasoning to recommend teaching practices targeted to groups of individuals who are similarly aged [5]. It is believed that this theory provides guidance for how members of another generation can understand and effectively reach learners of a particular birth cohort [4, 5, 10]. Some faculty members believe that students behave very differently from those in the past; [2] therefore, they face challenges in trying to communicate with a generation they may not fully understand [2, 10]. Most of the health sciences literature on the interface between generations and education is composed of theoretical and empirical articles, non-systematic, critical as well as non-comprehensive reviews [5, 11, 12] – with the majority of papers having focused on Millennial learners [10, 13, 14]. The Millennials (official name: Generation Y) are also called the ‘digital natives’ [15], the ‘instant messaging generation’, and until now, have become the most highly educated generation in western history [10, 13–15]. Scholarly and academically, they are defined as technologically literate and confident [10, 13–15].

Visual methods in research may include mechanical tools that can be used as data collection: such as photography, video, and film, or non-mechanical tools including drawings or other artworks. Visual methods have been considerably investigated in social sciences through studies on cultural significance, power relations and psychology research; [16–19] however, these concepts are still scarcely explored in teaching and research in higher education health sciences courses. Considering these positive attributes of visual-methods, we hypothesized that an investigation using drawings, and focusing on the subject *generations-theory* & *learning* could encourage thinking “outside the box”. This, therefore, would produce new ways of understanding and would help to disrupt well-rehearsed or planned responses by participants (as in case of written questionnaires or interviews, for example) [17].

Overall, the literature states that educational practices should be shaped according to the different generation cohorts [20, 21]. However, some authors have claimed that broad generalisations drawn from limited data are used as evidence to support instructional approaches, ignoring the internal differences and diversity inherent in any large group of people [5]. Considering these divergences and following our previous research studies that used visual data to inform and investigate educational practices [22, 23], this current study surveyed dental students and faculty members about their perceptions on the contemporary learning environment in higher dental education. Drawings were the data collected to qualitatively describe the voices of older generations (faculty members group) and younger generations (students‘ group) in regards to the current educational practices in undergraduate dentistry. This would help to understand perceptions from people born in different cohorts.

## Materials and methods

### Study design and instruments

This cross-sectional qualitative study is part of an umbrella project that collects data in the form of visual media (such as artwork, drawings, and mind-maps). The project was approved by the Behavioral Research Ethics Board from the university where data collection was conducted (ethical ID #2103; re-approved on 10/5/2021).

Participants were full-time dentistry faculty members (30%, 6/20) and Year-1 dental students (26.4%, 9/34) who produced artwork/drawings to graphically represent the current dental education environment. To analyze the drawing, two researchers used a multilayered process of thematic analysis to categorize the ‘feelings/opinions’ conveyed by the drawing. Under this approach, we aimed to give voice to faculty members and first year dental students to express their perceptions on the current learning environment.

### Context, environment and data collection

This study was conducted throughout March and April 2021. All Year-1 Doctor of Dental Medicine (DMD) students and full-time faculty members from the College of Dentistry at a university in Canada were invited to participate. The DMD program comprises four years of study in a lecture-based model with basic and clinical sciences disciplines. The Year- 1 students typically are enrolled in 10 courses, including basic sciences applied to dentistry (such as oral histology and embryology) and preclinical practice (such as operative dentistry). At the time of this data collection, dental students were approaching the end of their first year in the DMD program. Full-time faculty members are from a diverse of backgrounds in regards to training and nationalities. The instructional format is one faculty member teaching each discipline/course.

Data collection involved two stages of invitations that were conducted electronically by the College of Dentistry administration. Participants were invited to produce an artwork (drawing or painting) representing their view about the current learning environment. They were provided resources (three references) about using visual methods in research and were allowed to also use written text if they thought it might be necessary to explain their created artwork. The invitation also included a consent form document for review – the submission of the artwork (emailed to a person not involved in the research) implied the agreement to participate. To incentivize participation, and as a token of appreciation for participating, students and faculty received a small gift.

Since our dental school is small, and we expected a low number of participants, no demographic characteristics were individually collected to ensure anonymity within the relatively small number of eligible participants. To determine classifications for the two groups of participants, the individual who collected the artwork submissions (and anonymized them) provided the researchers with two groups of drawings. One group included all the students‘ drawings (they were Millennials and Generation Z), and the other group included all the faculties‘ drawings (they were Baby Boomer and Generation X). Researchers received the two groups of images but did not know in advance if the group belonged to students or faculties.

### Data analysis

Qualitative analysis was conducted by examining the student and faculty artwork depicting their feelings toward the current learning environment in dentistry. Two researchers independently analyzed the 15 artworks (students *n* = 9 and faculty members *n* = 6). Each artwork was analyzed in terms of what characteristics were elicited in the images (such as: positive or negative feelings/emotions, colors, and sizes) [23, 24]. For this purpose, emotions were defined not only as face or body expressions (physical dimension of the definition), but also as psychological dimensions in reaction to the outside world (*i.e*., expressing what is occurring inside the body to the outside world) [25, 26]. We conducted the analysis of the drawings with a critical theoretical approach using the following guiding questions “What is the main text/message conveyed by the images?” and “What are the counter-texts or the hidden (implicit) messages?” [23, 27]. We made efforts to analyse the images at a group level and not outlining conclusions about individuals. Our multilayered process of thematic analysis of the visual media (and written data, if any) was based on an interpretive approach, where codes, categories, and themes were then generated from the artwork independently for each of the two groups. We used an open/inductive approach [28] - codes were developed [29] directly from the artwork, without having a pre-set codebook. Coding types included, for example, “*negative affective*” (emotions/feelings) which tap into the inner cognitive systems of participants. Elements depicted in the artwork were directly used as codes, such as: ‘person without hands’ (drawing), ‘person without faces’ (drawing), “cameras on, please” (words), ‘patient’s legs are a rectangle and a triangle’ (drawing), and ‘a student attending the lecture in their own home” (drawing). These codes resulted then in the category “*no in person interaction*.” All different types of codes were collected into categories that were subsequently grouped to create themes [25] (these data are reported in Table 2).

**Table 2:** Codes, categories, and themes resulting from graphical data collected from the drawings, independently for dental students and dentistry faculty members.

	Codes	Categories	Themes
Group 1 (*n* = 9) students Millennials & Generation Z	- A big dental head loupe. *Inside the lens*: graphs; a maze; a DNA; chemistry formulas; medication/capsules; dendritic cell with happy eyes and smile; cell with organelles; syringe with anesthetic solution. *Inside the magnification lens*: a tooth with pulp and caries lesion, handpiece with bur, explorer, mirror, x-ray sign, tooth paste with fluoride, dental floss making a heart. - *Past*: students sit around a table and dialogue balloons (everybody talking at the same time). *Now (2021)*: woman at home (bedroom curtains) alone typing in a computer. - A man looking at the laptop screen (“Lecture” – word in the screen). - Thinking balloon about 2019 learning environment (professor in front of the blackboard & many students). - Notebook paper with the word “Two Halves”. - *Work*: typodont, handpiece, dental instruments, book, laptop, and a patient on the dental chair (with a bib). *Leisure*: ice rollers, car, video game control, gym weights, bottle of wine, kitchen appliances, a ship.	Contrast: basic sciences x dentistry applied subjects; current knowledge x future knowledge; preclinical x clinical; old x new; last year x current year; interaction X alone; work (top) X leisure (bottom)	- The dental learning environment is challenging because of the COVID-19 pandemic situation. There is loneliness, and lack of opportunities for empathetic interactions (the emotional reaction of sharing others internal experiences). - There is an evident division of basic and applied sciences. - We are diverse people. Time flies, everything passes by fast in today’s world. The future will arrive soon, and we will be dentists.
	- Student A, student B, student C and so on (on a zoom meeting) - Decreasing height, the tallest, the medium and the shortest. - Person a little bold with little bristly hair.	Differences in appearance	
	- One eye bigger than the other Facing back, struggling expression - Laptop on a table, online lecture, zoom meeting, empty keyboard, empty table, empty wall, camera ‘off’, empty windows, empty calendar (April) - Chat full of students talking: “Felling tired”, “Very tired”, “Isolate”’, “I look forward to meeting everyone in person”, “I miss in person classes” - 3 persons wearing personal protective equipment (PPE), one in the middle and 2 at each side - Persons without hands, persons without faces, face-shield, mask, long lab coats, scrubs, shoe cover - Colorful (a few colors)	Loneliness Negative feelings (missing people) Social distance Robotic feeling: strict biosafety rules	
	- Octopus, 8 arms *Inside the head light:* a landscaping, pine trees, smiling sun; music signs, hearts, ocean waves - *The headband of the loupe*: one side is an orthodontic appliance, and the other side is made of chat conversation balloons, question marks and explanation marks - A timeline - A calendar - Computer/smartphones/and other gadgets being concomitantly used - Through the window there is the moon - A coffee cup on the table - Little hair, does not look like a very young person	Multitasking No time to study (“anytime”) There is life outside school The future - a very specialized dentistry subject still unknown: orthodontics Aspirations in dentistry profession Time flies	
Group 2 (*n* = 6) faculty members Baby Boomers & Generation X	- Squares representing the learning environment (lectures & clinical setting) in different times - *Past:* a standing dentist with short sleeves and without gloves, patient on the dental chair with a serious face; - professor lecturing using an old projector with a laser pointing and a student taking notes using pen and notebook; patient in the chair (outdated chair, patient seated); dentist wearing a short sleeve lab coat - *Present:* a contemporary representation of the teaching/learning environment; a dentist wearing complete PPE looking at a computer screen with a patient image; professor lecturing at home using a laptop and a student attending the lecture in their own home; a tooth relating patient-dentist (arrows); dentist diagnosing through a computer screen; cellphone connected on Google; Zoom lecture - Work (on the top) X Leisure (on the bottom) - Work: book, computer, cellphone - Leisure: Snapchat, Instagram, Twitter, WhatsApp, You Tube & Facebook - Patient’s ‘body’ is a square with an apple fruit (Apple logo) and a red heart - The chair seat has divergent arrows - Patient’s legs are a rectangle and a triangle - The foundation of the chair is a plastic tooth (or extracted tooth) included in a preclinical resin block	Contrast: past x present; old x new foundational is not clinical (the base is an isolated fake tooth) x tele-dentistry (contemporary) is ‘clinical’	- The dental learning environment is challenging because of the COVID-19 pandemic situation. However, as the positive, compassionate, and supportive feeling remains amongst people in these difficult times, the environment is pleasing and worthwhile. - There is an evident division of basic and applied sciences
	- Flowers are passing as a gift (hands holding flowers and leaves), lily rose, daisy - Creative, artistic - A tooth relating patient-dentist (arrows) - A table with a laptop and a cellphone, multi-connection - Apple logo and a red heart (patient) Dentist without strict PPE	Positive Supportive	
	- Patient’s legs are a rectangle and a triangle - Dentist diagnosing through a computer screen - A dentist wearing complete PPE looking at a computer screen with a patient image	Challenges in our current education Connection between preclinical and clinical	

## Results

Qualitative analysis from artwork data (*i.e*., the codes, categories, and themes identified in the drawings) was then undertaken to know student and faculty perceptions about the learning environment in the DMD program.

We observed that drawings for both groups represented positive and negative features. As examples, some graphical representations (or written text) collected from the student artwork depicting negativity included: one eye bigger than the other (struggling expression), person alone studying and working from home, “isolated” (word), an empty keyboard, an empty table, an empty wall, empty windows, an empty calendar (no social events), no in-person meetings etc. Some of the categories generated from these codes consisted of ‘loneliness’ and ‘social distance’ (find more information on negative and positive features expressed by students and faculty members in Table 2).

**Fig. 1:**
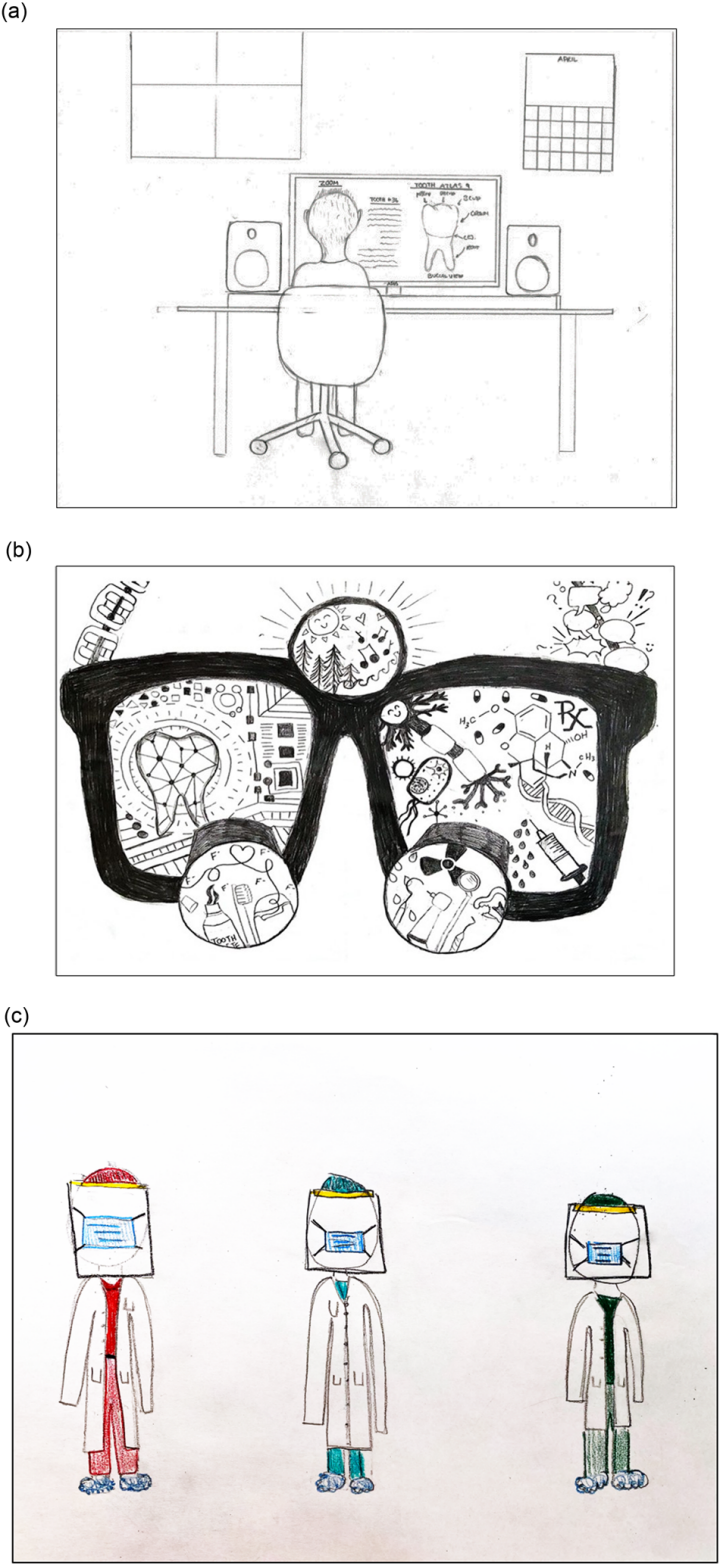
Three artwork created by Year-1 dental students (Millennials and Generation Z) showing some features illustrating the three *Themes* for this group (see Table 2). *Theme 1*: student studying on their own at home (**a**), and lack of touching between friends and the required social distance (**c**). *Themes 2 and 3:* (**b**) basic and preclinical knowledge inside the glass lens, which is represented by chemical formulas, a neural axon, graphs, a diagram-tooth, sodium hypochlorite syringe etc., with these graphics also indicating ‘*the present’*; and clinical dentistry inside the magnification lens, which is represented by tooth paste, dental floss, fluoride symbols, radiology symbols, dental handpieces with burs, a cavitated tooth and a heart (probably a caries diagnosis and management situation), with these graphics also indicating ‘*aspirations for the future*’; unique hair and ears of a person at home (**a**), and distinct heights and hairstyles of three classmates (**c**).

**Fig. 2:**
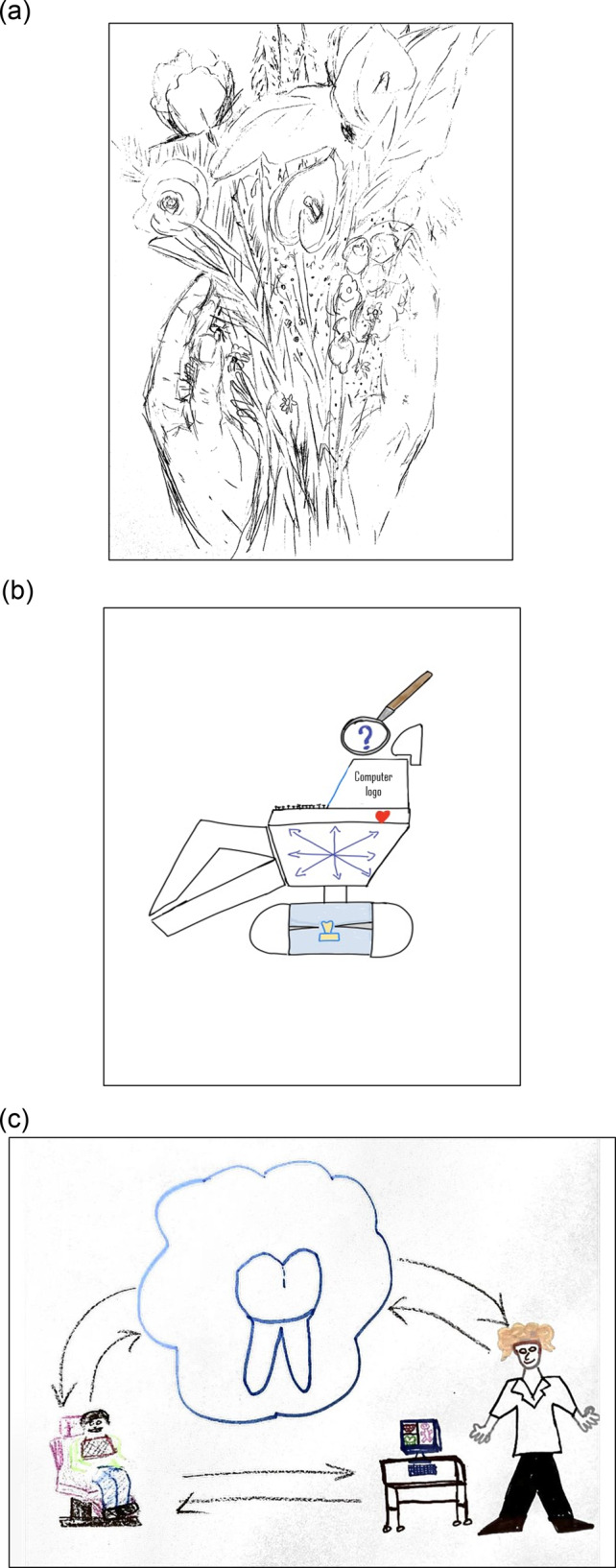
Three artwork created by dentistry faculty members (Baby Boomers and Generation X) showing some features illustrating the two *Themes* for this group (see Table 2). *Theme 1*: Image (**c**) a patient sitting in an outdated chair, (rather than lying down), an old computer model, a dentist wearing short sleeves lab coat, no gloves, absence of head loupes and relatively long and loose hair; evokes the positive traditional care and patient-dentist interaction (both persons are smiling). Along with this, the distance between patient and dentist (each one in one corner of the drawing) seems to make the diagnosis and treatment planning more challenging (the big tooth and arrows minimizing the gap for a comprehensive care) – which reminds the challenges caused by the pandemic situation nowadays. A positive, compassionate, and supportive feeling remains amongst people in these difficult times, with an environment that is pleasing and worthwhile: image (**a**) contains hands holding flowers that appear to be a gift. *Theme 2*: Image (**b**) contains a tooth mounted in a typodont as a base for the dental chair, suggesting the preclinical setting, one of the bases of clinical dentistry. The small red heart reminding the patients. The loupe with a question mark, remembering that there are multiple ways (arrows on the computer screen) to investigate the patient’s diagnosis.

The themes generated from codes and categories indicated that both groups of participants expressed perceptions in relation to the evident division of basic/preclinical and applied/clinicals sciences (this model of education implies that learners should first learn basic and biomedical sciences and then move to clinical sciences) [30]. Other perception that seemed aligned between groups was the idea about the challenges caused by the pandemic situation. Our complete sample of students depicted some representation of feeling lonely and missing personal interactions (representations linked to the COVID-19 pandemic restrictions in place in 2021). Faculty members also indicated that the contemporary dental education environment is difficult, correlated present and past, but they showed hope to see the situation get better. Students‘ drawings evoked the following topics: digital connectedness, diversity, time goes by, and future aspirations in Dentistry. Figures 1 and 2 show more of these representations/features that produced the themes.

## Discussion

Drawings were considered a valuable tool in giving voice to participants regarding the contemporary learning environment that dental students and faculty members presently interact within. Amongst the themes resulting from our qualitative analysis of drawings for both groups, were included: the challenging situation caused by the COVID-19 pandemic and the awareness about the division between basic/preclinical and applied/clinical courses. The findings from this study may be useful as evidence to understand better the similarities and differences in young dental students‘ and faculties‘ perceptions; and to help faculty members evaluate the appropriateness of their own teaching practices.

Even though the two groups of participants came from different generations [Year-1 dental students were Millennials (born between ~ 1981 and 2000s) and Generation Z (born between ~1997 and 2012), and faculty members were Baby Boomers (born between 1946 and 1964) and Generation X (born between 1965 and 1980)], they depicted some similar/aligned ideas. This is interesting because looking at different generations cohorts of people help us to understand shifts in socio-cultural-education environment [2, 31]. More information about these outputs can be found in Table 2 and Figs. 1 and 2.

Although our participants did not show evidence that they would prefer a more integrated curriculum, the researchers found that representations of the interfaces between laboratory-clinic suggested their perceived difficulties to connect these stages of education. The poor connection between preclinical and clinical actions is a well-discussed topic in health education literature [32]. It is well-known that predicting future professional achievement has been an elusive goal in dental education [33]. Quantitative studies proved that most measures used to predict achievement in preclinical and clinical activities have shown weak predictive values [33–35]. One study found no correlation between a typodont preparation examination, designed to provide a measure of students’ clinical skills, and a clinical competency exam involving the preparation of a full crown in a patient [33]. In addition, some of our previous qualitative findings have found that dental students were skeptical of the usefulness of learning procedures on mannequins/typodonts and how this experience would translate to making them proficient enough to provide dental care for a real person [23]. The traditional dental education context consists of very different preclinical and clinical conditions, in which students acquire various skills sets necessary to be successful [36]. The preclinical simulation courses usually include didactic content and a procedure demonstration, followed by students performing the assigned skill at their stations. Then, students perform the skill and, at the end of the session, show the outcome—or product—to the instructor for feedback [37]. This model leaves important skills unaddressed, such as communication, critical thinking, decision making, and management. It is known that the repetition of procedures by the preclinical student or the grades received in preclinical practical exams do not guarantee clinical competency. Experiences students are faced with when entering the clinics are not encountered in the consistent and tightly controlled traditional preclinical environment [36, 38].

Our current findings made us to suppose that the over-focus on psychomotor skills in the laboratory courses enhanced the perceived division between preclinical and clinical dental education. It is important to highlight that our reflexive approach to this discussion is grounded on the context that we live, professional and emotional self, social and cultural identity, unconscious bias and motivations, and conscious reactions and responses [39]. We are aware that our approach did influence the interpretation of our data; but we made efforts to not have preconceived notions about the outcomes. Both assessors were trained under the traditional dichotomy between preclinical and clinical dental education, which was extensively experienced during our time as students (we are from Generation X), and we currently work in a specialty-based traditional model. Therefore, we embraced that our own experiences influenced our dialogues in this context, because this is an important way to produce co-created knowledge and to take advantage of collaboration between the researchers and the research subjects [39–42].

The challenging situation caused by the COVID-19 pandemic was frequently depicted in both groups of participants‘ drawings. Student’s artworks expressed their dissatisfaction about the current learning environment and that they missed daily moments of having interpersonal interaction. These findings were easy to understand when we linked them to the context and time of this study (data collection was conducted in early 2021, when the COVID-19 pandemic restrictions were in place). Our sample of year-1 dental students, since entering the dental program, had received most of their dental education remotely, with a few encounters in the preclinical lab. Also, the few opportunities to have personal encounters with classmates and faculty were marked by the need to social distance (creating not only physical distance, but also emotional gaps). Faculty members also expressed concern in relation to the current learning environment [2, 33], making their voices aligned with the students‘ voices. Some of the graphical data representing the current situation included: a dentist diagnosing through a computer screen, and a dentist wearing complete PPE looking at a computer screen with a patient image (that could be interpreted as ‘the technology in between them’). Despite that, we also detected positive graphical data, such as: a heart, smiles, connecting arrows, hands, flowers etc.) (please see Group 2’s codes/graphics in Table 2) depicting that a supportive feeling and optimism remains amongst people.

Students‘ drawings evoked the ideas of ‘digital connectedness’, ‘diversity’, ‘time goes by’, and ‘future aspirations in Dentistry’. Millennials are defined as technologically literate, confident, motived by self-interest, team-oriented, assertive, and present with emotional intelligence [21, 43]. Millennials want to know what is expected from them in explicit terms and expect direct feedback [10, 21, 43, 44]. Generation Z‘ students (sometimes are referred as iGen or Centennials) [45–47] are the most diverse generation in modern history, and its members are attentive to inclusion across race, ethnicity, sexual orientation, and gender identity [47, 48]. The contemporary higher education has increasing the number of diverse races; as well, students may be from lower-income households and may have parents who have not attended college [45, 47]. This is a result of equity approaches. Generation Z learners are less self-directed (compared to Millennials) and need innovation (communication through technology), personalized services (from career development to tutoring to mental health), and equitable opportunities for engagement [45, 47]. Grounded on our analysis, codes depicting diversity included different hair styles and colors, different peoples‘ heights, and different genders/non-gender. The sensation that time goes by quickly while they are spending the majority of their time studying, made students think about their future aspirations in dentistry. Some codes depicting this idea included the day-night appearance through the window, computer/smartphones/and other gadgets being concomitantly used by students, a timeline, a calendar etc. Our insight about those graphics was that students may adapt to different and constantly changing environments [10, 45–47]. Educational approaches to engage these cohorts would be short theoretical lectures, rethinking the need for repetitive procedures to guarantee proficiency, and being flexible. It is known that not every person in a generation may be representative of the traits of the cohort; thus, diverse teaching styles and diverse approaches may be beneficial to reach this ‘miscellany’ of learners. Faculty members may need to carefully assess each of the individuals in class, which would facilitate to personalize educational approaches.

This study had weaknesses and strengths. It was powerful tool to reinforce the validity of visual methods as an approach in research, and to distinguish several actual graphical features (features that might be intentionally or unintentionally represented in the drawings) that gave voice to participants. These voices may have been invisible in more traditional qualitative approaches, such as interviews or questionnaires. A weakness would be the manual coding of qualitative data. Manually coding would include the coder’s cognitive biases and can influence the coding process [49]. However, the drawings provided us with already discernible and ‘pre-coded’ data (graphics), and from time to time, we checked one another’s coding (and resolved the disagreements) - these may have minimized cognitive biases. It is important to remember that qualitative research is different from quantitative, in QUAL analysis, rather than trying to eliminate all bias, the aim is to acknowledge areas which are more open to interpretation by the researchers [49]. An additional limitation of this study, which is applicable for other ‘visual/drawings’ approaches, is the low level of agreement to participate. Many individuals who are attracted to the dental field are not accustomed to producing artwork and are, consequently, afraid of their possible ‘lack of dexterity’ for drawing or painting. This current study had low compliance (26.4%, 9/34 students and 30%, 6/20 faculty members), which created challenges for the researchers in producing expressive results and weakened the possibility of external transferability. Despite our small sample size, our results may be useful to spread the novel idea of using illustrations in allowing participants to express their emotions in education and learning.

## Conclusions

Drawings produced by first year dental students (younger generations) and faculty members (older generations) were unique, innovative, and an interesting tool to express their voices regarding today’s dental education learning environment. Although the two groups of participants came from different generation cohorts (students were Millennials and Generation Z; faculty members were Baby Boomers and Generation X) they similarly perceived that dental education is going through a challenging time, because of the restrictions caused by the COVID-19 pandemic; and they were aware about the evident division between basic/preclinical and applied/clinical courses. Faculties showed hopeful signs that the situation may get better. Students‘ drawings evoked the following topics: digital connectedness, diversity, time goes by, and future aspirations in Dentistry. Using drawings in higher dental education research can help educators to gain additional insights into the perceptions of their students; the drawings provide a supplemental method to understand their opinions on a specific topic. These insights can consequently help educators to personalize teaching approaches to better meet the needs of the students.

## Supplementary information

Figure 1

Figure 2
