# Supplementary figures and images for: Drawings to explore faculties‘ and students‘ perceptions from different generations cohorts about dental education: A pilot study
Source: BDJ Open. 2022 Jun 15;8:17. doi: 10.1038/s41405-022-00109-5 (PMC9199317; doi:10.1038/s41405-022-00109-5)

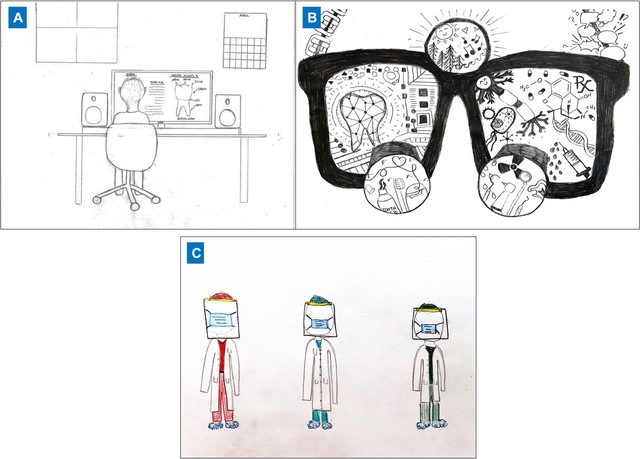

Supplement: Supplementary file 1 — Figure 1 [file 41405_2022_109_MOESM1_ESM.jpg]

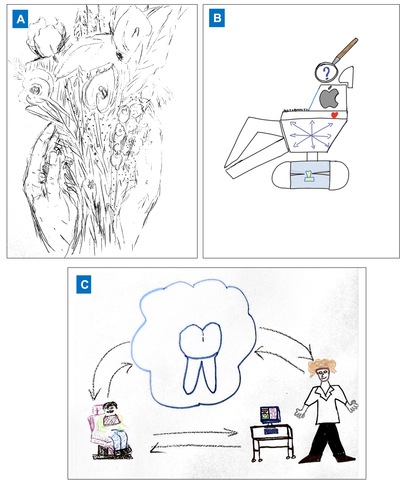

Supplement: Supplementary file 2 — Figure 2 [file 41405_2022_109_MOESM2_ESM.jpg]
